# Tuberculous Osteomyelitis of the Left Fifth Metatarsal and Phalanx Without Pulmonary Involvement: A First-of-Its-Type Report

**DOI:** 10.7759/cureus.40737

**Published:** 2023-06-21

**Authors:** Sankalp Yadav, Gautam Rawal, Madhan Jeyaraman

**Affiliations:** 1 Medicine, Shri Madan Lal Khurana Chest Clinic, Moti Nagar, New Delhi, IND; 2 Respiratory Medicine and Critical Care, Max Super Speciality Hospital, New Delhi, IND; 3 Orthopaedics, ACS Medical College and Hospital, Dr MGR Educational and Research Institute, Chennai, IND

**Keywords:** cbnaat/ xpert/ rif assay, tuberculosis, proximal phalanx, fifth metatarsal, tuberculous osteomyelitis

## Abstract

Tuberculosis manifesting at extrapulmonary sites is relatively rare. Isolated cases involving small bones of the foot are the rarest of the rare. We herein present the case of a 30-year-old Indian female who presented with pain and a wound with a discharging sinus over her left foot. In the absence of constitutional symptoms of tuberculosis, this case was diagnosed as isolated tuberculous osteomyelitis of the left fifth metatarsal and phalanx with the help of radiography, a cartridge-based nucleic acid amplification test, a line probe assay, a culture of the biopsy specimen, and magnetic resonance imaging. She was initiated on anti-tubercular treatment per national guidelines.

## Introduction

Tuberculosis is a major concern for public health [1]. This bacterial disease is caused by *Mycobacterium tuberculosis* and can occur at extrapulmonary sites like the bones and joints [2]. Isolated cases of extrapulmonary tuberculosis in bones and joints are available in the literature where no pulmonary involvement was reported.

With about 2.2-4.7% incidence of tuberculosis of bones and joints in developed countries, this type of tuberculosis contributes to about 10-15% of total extrapulmonary tuberculosis [3]. These numbers increase to 15-20% in high-burden countries [3]. Foot and ankle tuberculosis make up about ten percent of total osteoarticular tuberculosis [4]. The incidence of metatarsal tuberculosis is <0.5% [5].

Tuberculosis of the bones of the foot is a relatively infrequent occurrence [6]. Isolated cases with no pulmonary invasion by the bacteria are extremely rare. We herein report a case of tuberculous osteomyelitis of the left fifth metatarsal and phalanx in a patient who came with complaints of pain and discharging sinuses from the fifth toe of her left foot. A detailed diagnostic workup backed with radiometric techniques helped in the diagnosis, and she was started on four-drug anti-tubercular treatment per her weight.

## Case presentation

A 30-year-old non-diabetic Indian female belonging to a low-income family reported to us with major complaints of pain, swelling, and a wound with a discharging sinus over the left fifth toe for two months. This was accompanied by a yellow-colored, non-foul-smelling, purulent discharge for two months. It was difficult for her to bear weight on the left foot, and this was associated with an apparent limp.

She had a history of trauma three months ago when a heavy wok fell on her left foot's lateral aspect while cooking. She did not consult an orthopedic facility at that time and continued to consume over-the-counter drugs like nonsteroidal anti-inflammatory drugs (NSAIDs). She reported to our center only once the pain became unbearable and a discharging sinus developed. The pain was sudden in onset post-trauma and had increased over two months. It was constant, mainly in the left foot, and increased with movement. There was no history of classical symptoms of tuberculosis or any contact with a tuberculosis patient, and there was no remarkable past medical or surgical history.

A general examination revealed an average-build female with a pulse of 76 per minute, a temperature of 98.4 degrees Fahrenheit, a blood pressure of 112/78 mm Hg, a respiratory rate of 17/minute, and oxygen saturation (SpO2) of 99 percent in room air.

**Figure 1:**
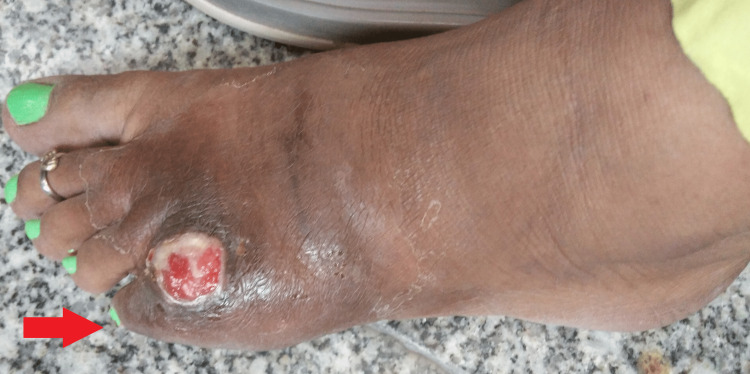
Swollen left fifth toe with a wound and an active discharging sinus

Local examination revealed a swollen left fifth toe with a 5 X 5 cm wound and an active discharging sinus over the dorsum of the left fifth toe with clear edges (Figure 1). There was an incommodious range of movement of the fifth metatarsophalangeal joint due to excruciating pain. There were restricted eversion and inversion movements of the affected foot, with painful dorsiflexion and plantar flexion. The abutting skin was comparatively dark, flaky, and warm to the touch, but there were no dilated veins. Other joints in the left foot were normal. Further, there were no similar observations on the right foot. Furthermore, there was no cyanosis, koilonychia, lymphadenopathy, clubbing, pallor, or icterus. Her systemic examination revealed no remarkable issues.

This initial workup was suggestive of a trauma-induced wound; therefore, a preliminary diagnosis of tuberculous osteomyelitis of the left fifth toe was made with differentials for fungal osteomyelitis and pyogenic osteomyelitis.

**Figure 2:**
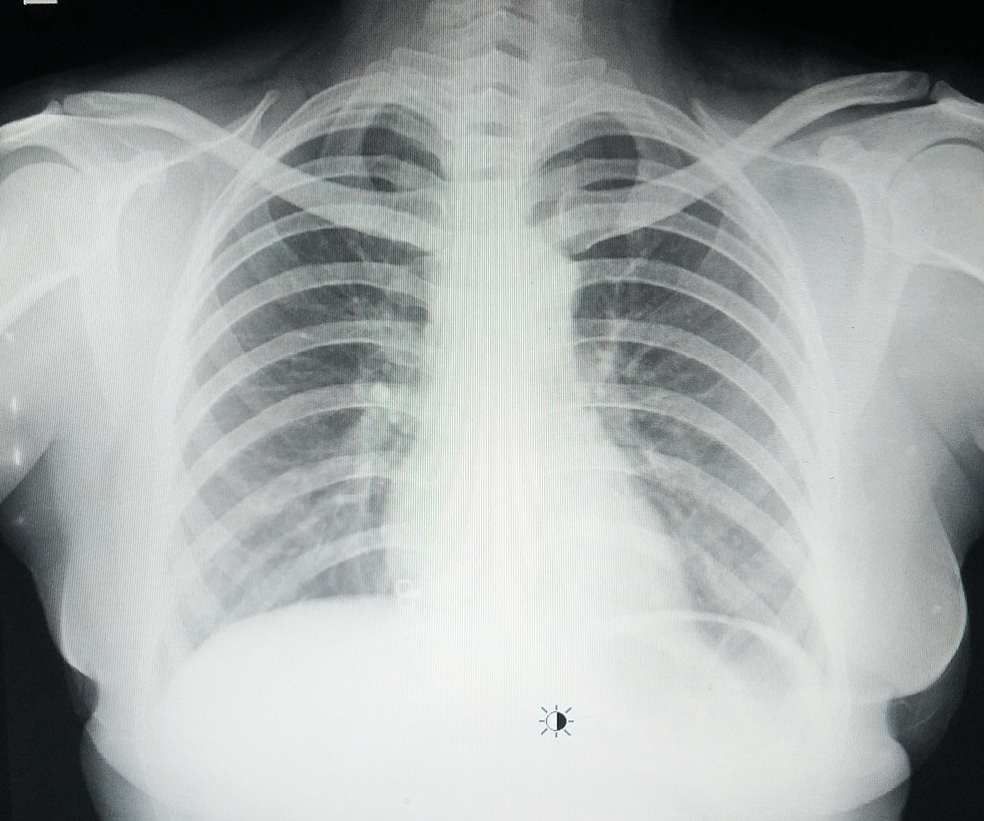
A normal plain chest radiograph antero-posterior view

A detailed laboratory workup revealed normal serological indicators except for an augmented erythrocyte sedimentation rate of 51 mm in the first hour and a raised C-reactive protein value of 8 mg/l. Her HIV was non-reactive; a tuberculin skin test, an interferon gamma release assay, and a rheumatoid factor test were negative. A respiratory workup involved an induced sputum test for acid-fast bacilli and a cartridge-based nucleic acid amplification test (CBNAAT) of the same, but all were negative. A plain chest radiograph was not suggestive of pulmonary tuberculosis (Figure 2).

**Figure 3:**
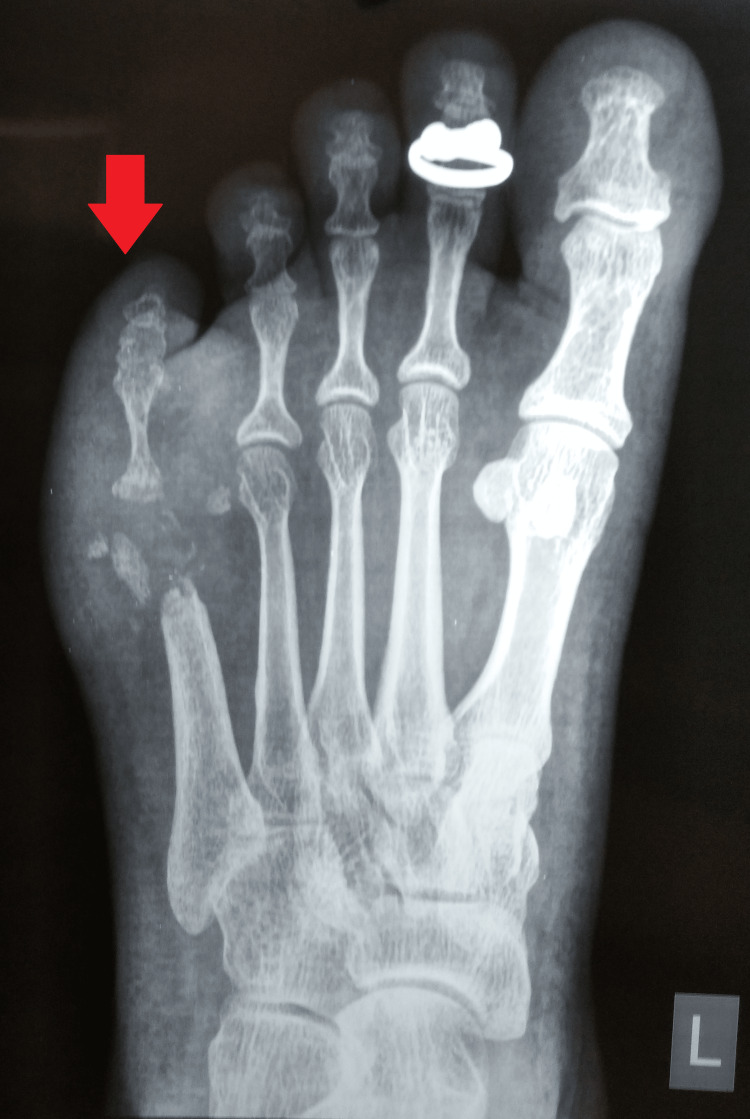
Antero-posterior radiograph of the left foot

**Figure 4:**
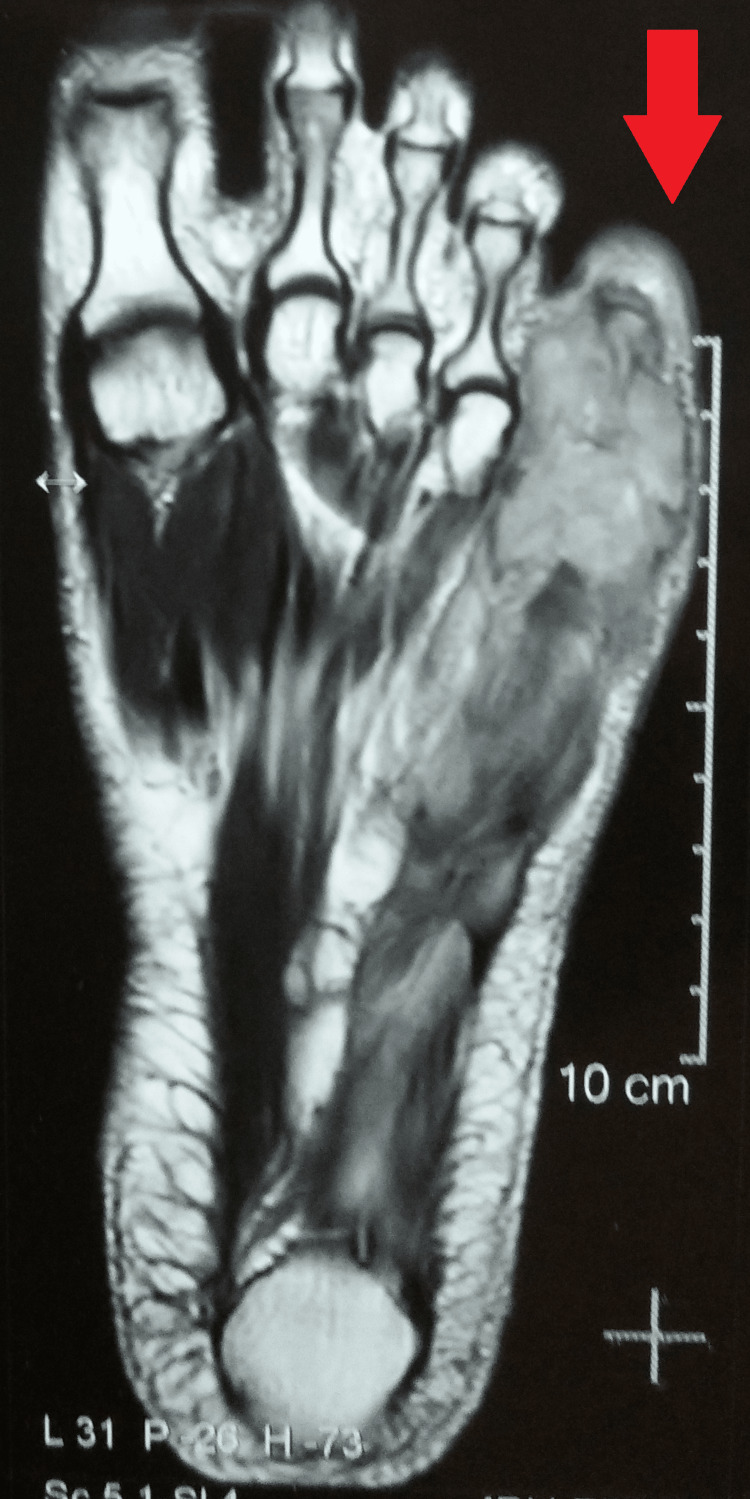
A MRI of the left foot showing the involvement of the distal fifth metatarsal and proximal phalanx MRI: magnetic resonance imaging

Antero-posterior and oblique radiographs of the left foot were suggestive of a destroyed distal fifth metatarsal and proximal phalanx with adjacent soft tissue swelling (Figure 3). This was followed by magnetic resonance imaging (MRI) of the left foot, which was suggestive of significant cortical destruction of the distal fifth metatarsal and proximal phalanx, with a large collection about 51 X 25 mm on the dorsal aspect of it surrounded by significant subcutaneous tissue edema (Figure 4).

An open biopsy and wound debridement were done, and nearly 10 ml of yellowish-colored pus were removed. The samples were sent for histopathological and relevant microbiological tests. The smear for acid-fast bacilli from the aspirated pus on Ziehl-Neelsen staining was negative. However, histopathology was indicative of tuberculosis with caseating necrosis and epitheloid granulomas with lymphocytes.

The CBNAAT of the pus revealed a low detection of *Mycobacterium tuberculosis* with sensitivity to rifampicin. A supplemental sample was sent for line probe assay (for drug-susceptibility testing for rifampicin and isoniazid resistance) and culture to the national reference laboratory. *Mycobacterium tuberculosis* was detected on a line probe assay (no rifampicin or isoniazid resistance and no mutations in rpoβ, inhA, and katG genes) and grew on liquid culture media BACTEC (Becton Dickinson and Company, Franklin Lakes, New Jersey, United States) with sensitivity to the first-line anti-tubercular drugs.

A final diagnosis of tuberculosis of the fifth metatarsal of the left foot with a phalanx without pulmonary seeding was made. She was advised anti-tubercular treatment per her weight initially with quadruple drugs (rifampicin, pyrazinamide, ethambutol, and isoniazid) for 56 doses in the initiation phase, which was followed by a three-drug therapy (rifampicin, ethambutol, and isoniazid) for the next 10 months, per the national guidelines.

Additionally, a tablet of pyridoxine was also given for the entire treatment duration. Also, she was advised on her diet by a counselor to eat a diet rich in proteins. An orthopaedician was consulted, who advised continuing the conservative management and surgical intervention after 12 months with a regular follow-up. She and her husband were also reluctant to undergo any surgery.

Presently, she is doing well, with a slight improvement in her discharge from the wound and no major adverse drug reactions. After one month of treatment, at her request, she was transferred to a different district.

## Discussion

Tuberculosis of bones and joints is a very seldom reported entity and usually represents one to three percent of total cases of tuberculosis [3]. Mostly seen at sites like the hip, spine, knee, and bones of the foot [7]. When the disease is without the involvement of joints, it could manifest in the metatarsals, sacroiliac, sternum, ribs, metacarpals, skull, pelvic bones, and scarcely the large tubular bones [7]. Tubercular involvement of the foot is extremely rare and mainly seen in the calcaneum, talus, first metatarsal, navicular, medial and intermediate cuneiforms in descending order [8].

The disease is often associated with a delay in reporting, which adversely impacts the outcomes of its management [6]. The usual causes of these delays are subtle and confusing presentations of the disease, a lack of classical symptoms of tuberculosis, the paucibacillary nature of the disease, and a lack of treatment awareness among the treating physicians, which makes it a difficult diagnosis [9].

The commonest source of spread of *Mycobacterium tuberculosis* in such cases is hematogenous [3]. This disease has a female proclivity and is mainly seen in patients in their second or third decade of life [10]. Pulmonary involvement is often reported in about 1/3 to half of the cases, and thus a detailed workup to rule out concomitant pulmonary involvement should always be made [3].

Clinical features are atypical, like pain, swelling, and discomfort while walking, which are often indistinguishable from other musculoskeletal disorders [7]. However, certain presentations like painful swelling of the involved joint or bone with an insidious onset, a non-healing ulcer, and a sinus associated with a discharge should alert the treating clinicians [11].

Radiography is non-specific in tuberculosis of the bone and joints [3,7]. Commonly seen features on a radiograph could be either lytic lesions, joint effusion, periarticular osteoporosis, bone marrow edema, soft-tissue collections, or tenosynovitis [7].

The management is mostly conservative with anti-tubercular chemotherapy [6,12]. However, surgical intervention is usually sought in cases where the disease is intractable or as a limb salvage method in cases with deformities in the hindfoot joints [12].

There are a few reports of tuberculosis of the metatarsal and phalanx separately, but there is a paucity of data where isolated involvement of both bones without pulmonary seeding is present [13]. Besides, a detailed search revealed that isolated fifth metatarsal and phalanx tuberculosis without concomitant pulmonary involvement has never been reported anywhere in the world.

In a study by Dhillon et al. on 92 patients with foot tuberculosis, metatarsal and phalangeal tuberculosis were seen in only three cases each [14]. Mittal et al., in their 44 patients, found metatarsal and phalangeal involvement in 18 and 15 cases, respectively [15]. Another study by Agarwal et al. on 21 pediatric patients revealed tuberculosis of the fifth metatarsal in only two cases [16].

Overall, we presented an interesting case where the concomitant involvement of the fifth metatarsal and phalanx is reported without pulmonary involvement. It is emphasized here that the paucity of data related to such presentations should encourage the reporting of similar cases, especially from high-burden countries.

## Conclusions

Tuberculosis of the small bones of the foot is a challenging diagnosis and requires a high index of suspicion. These cases often report late, and this could ultimately impact the management. It is imperative that paucibacillary cases like the present one be evaluated in detail with culture, histopathology, and radiometric techniques. Prompt anti-tubercular chemotherapy should be initiated to prevent the spread of the bacteria and further deterioration of the bones.
